# Book review of “Biomagnetics: Principles and Applications of Biomagnetic Stimulation and Imaging” edited by Shoogo Ueno and Masaki Sekino

**DOI:** 10.1186/s12938-016-0170-5

**Published:** 2016-05-12

**Authors:** Pere J. Riu

**Affiliations:** Department of Electronic Engineering, Center for Research on Biomedical Engineering, Universitat Politecnica de Catalunya, Barcelona, Spain

## Abstract

This article is a review of the book “Biomagnetics: Principles and Applications of Biomagnetic Stimulation and Imaging” (ISBN 978-1-4822-3920-1, 343 pages) edited by Shoogo Ueno and Masaki Sekino published by CRC Press, Taylor and Francis Group, LLC in 2015. The content of the book and its importance for biomedical engineering have been discussed in this invited review.

## Book review

The book titled “Biomagnetics: Principles and Applications of Biomagnetic Stimulation and Imaging” edited by Shoogo Ueno and Masaki Sekino has 343 pages and is divided in 10 chapters. It Contains a comprehensive description of applications used for medical diagnosis or research purposes involving the use of magnetic fields, including brain stimulation, measurement of magnetic fields produced inside the brain, and source location methods; and different modalities of MRI imaging. The book also includes therapeutic applications and the description of a speculative model of interaction between magnetic fields and biological matter; a chapter on safety aspects and a final one describing future applications of magnetic fields in biology and medicine.

According to the authors, the core of the book is based on lecture notes for graduate students in the Electronic Engineering and the Biomedical Engineering Departments at the University of Tokyo. It is then expectable to find broadness in scope but also rigor in the presentation and a solid biological and electromagnetic foundation. Readers without a good background in electromagnetics may found it difficult to follow in some parts.

Shoogo Ueno is one of the parents of the modern biomagnetics discipline, making significant contributions since the 1970s. He developed the figure-8 coil in late 80’s, which opened a new era in magnetic stimulation by permitting a much higher spatial resolution than conventional coils. S. Ueno retired from the University of Tokyo as Professor Emeritus in 2006, and is currently a professor at the Kyushu University. Masaki Sekino earned his Ph.D. in 2005 and has followed Prof. Ueno’s steps, taking them to novel applications such are MRI imaging of currents or impedance distribution.

## Specific content

Chapter one contains a brief review of the historic evolution in the use of magnetic fields for therapeutic or diagnostic applications, providing a nice view of the very broad range of frequencies and amplitudes of the magnetic fields involved in biological applications, from stimulation at low frequencies and high amplitudes down to measurements performed with SQUIDs, or hyperthermia in the radiofrequency range.

Chapters two and three deal with transcranial magnetic stimulation (TMS) and its variants. The principles of neuronal stimulation by magnetic fields are explained, as well as details on the electronic circuits and coils used in medical applications. Therapeutic applications for pain relieve or depression are discussed, and also studies for the application of TMS to other neurological diseases. Research application to assess cognitive function are introduced as well.

Chapter 4 explains the measurement of weak magnetic fields produced inside the human body, and specifically inside the brain. Magnetoencephalography (MEG) using superconductive quantum interference devices (SQUIDs) is described, as well as algorithms for the geometrical location of the currents sources that produce the measured fields.

Chapter 5 and 6 are devoted to MRI. The principles of operation and the imaging modalities used in clinical MRI are presented. In addition to classical approaches, more recent techniques such are diffusion tensor imaging or functional MRI (fMRI) based on blood oxygenation level dependence (BOLD) are also introduced. Newer applications of MRI that enable us to image the distribution of electrical currents inside the body, or to map electrical conductivity, are introduced and the possible clinical relevance is discussed.

Next chapter is devoted to effects of magnetic fields at cellular level that may lead to cell destruction or cell growth promotion. Applications for cancer control or tissue healing are discussed.

Chapter 8 presents a new model for the interaction of magnetic fields with biological material. Theoretical foundations and some preliminary experimental results are presented and discussed.

Chapter 9 is devoted to safety. Interaction mechanisms are introduced and exposure guidelines, standards and legal actions to ensure patient’s and worker’s safety are presented.

Last chapter presents the latest advancements on the field including, but not limited to, molecular imaging or coil array designs to achieve more spatial resolution in both measurement and therapy.

## Book strengths

The interactions between (electro)magnetic fields and biological materials are complex. Complexity increases when biological materials are alive and performing a physiological function. Understanding these interactions require solid knowledge on electromagnetism, biology and physiology, and an openness of mind. The book succeeds in presenting this complex matter in a rigorous but understandable way. This book will be of great interest for both engineers and physicist working in the fields, as well as for the technically-skilled medical doctors. As a word of caution, those without some knowledge of electrodynamics can be somewhat frustrated. It is clearly not a book intended for mass divulgation, but the reading is not difficult if he reader has some background on the matter. The final chapter, on new horizons, will be worth of reading by those seeking a research topic in that field.

## Book weaknesses

Concentrating all the knowledge on bio(electro)magnetics in 343 pages is certainly not easy. However a book that will be mostly regarded as a reference on the field, and will probably be used as a teaching material for graduate courses should be more careful with oversimplifications that are later much difficult to remove from people’s minds. E.g. JC Maxwell never derived the four equations usually known as “Maxwell’s Equations”, they were formulated by O Heaviside in 1884. Or the idea that electric fields are induced in biological tissues because they are conductive: electric fields are induced irrespective of the conductivity of bodies. This idea is perpetuated throughout the book, and sometimes it is stated that electric fields are induced, while some others eddy currents are said to be induced (eddy currents of course depend on conductivity). Or the wrong idea introduced in Chapter 4 that quasi-static approximation of Maxwell’s Equations means that both temporal derivatives of fields are zero. The usual definition is that one derivative, but not both, is zero.

The use of mathematical formalism must be well balanced. The depth and extension in the use of mathematical expressions seem to be a bit arbitrary. Some sections have detailed derivations of formulas, while others present only a handful of expressions that seem to come from nowhere. E.g. the 8-shaped coil, that is a key improvement on TMS, is described in a very naïve way, while the electric circuits used to excite the coils are described at very low level by differential equations. A similar thing happens with the explanation of physiological functions, some of which are explained with an unnecessary detail, e.g. the action potentials, while others are just mentioned. This adds difficulty to the reader without a solid background.

The last chapters of the book contain somehow speculative applications. E.g. the imaging of currents or conductivities by means of MRI is far from being a usual procedure, and the practical limitations are not yet known. One of the toughest is the poor knowledge on the expected dielectric properties of biological tissues in vivo and in situ, since almost all data available comes from excised tissues. The model presented in chapter 8 is also highly speculative and experimental data presented imply the need of strong fields and concentrations of ferritin also much higher than those encountered in human bodies. Although this fact is acknowledged by the authors, its introduction departs a bit from the rest of the book, where most of the applications presented are well founded theoretically and clinically.

The chapter on safety comprehensively reviews exposure guidelines and the new European Directive for the safety of workers. It is a pity that only MRI related applications are covered. Other clinical procedures, like TMS are known to produce fields that can surpass action values for workers as defined in the 35/2013/EU Directive. In addition, it would have been fair to acknowledge the pioneering work of ANSI/IEEE C95 set of standards, and the current work on IEEE ICES SCC28 Committee, in addition to ICNIRP.

The quality of figures that contain images deserve a specific comment. All images are in grayscale. This fact alone would, sometimes, make it difficult to see the features mentioned in the text. But in addition, the quality of the printing is bad, making most of them useless. While this fact is not attributable to the authors, it certainly decreases the value of the book.

## Conclusion

This is a highly recommended book, despite the mentioned weaknesses. Anyone working or wishing to work in the filed should read it. In that multidisciplinary field, broadening the scope in a rigorous way is the only way to improve the knowledge of the specific application that anyone may be working on.

## A final word on the title

The title of the book is “Biomagnetics”. In addition the terms like “biomagnetic field” or “biomagnetic stimulation” are used (and in my opinion, abused) with profusion in the text. A magnetic field produced by a coil is quite insensitive to the fact that it is produced close to a biological material. Conversely, a magnetic field produced by a current inside the body is identical to a magnetic field produced by a current flowing in an electronic circuit. In addition, for most of the applications, the magnetic field is just a mediator to induce electric fields or mechanical forces to non-biological particles. MRI is one notable exception where applied magnetic fields directly interact with magnetic moments in the matter, biological or not. In my opinion, “biomagnetics” can be used to describe the discipline, but other terms and uses should be avoided, to prevent misunderstandings.

